# *MYH11* Suppresses Colorectal Cancer Progression by Inhibiting Epithelial-Mesenchymal Transition via *ZEB1* Regulation

**DOI:** 10.32604/or.2025.063501

**Published:** 2025-08-28

**Authors:** Yuhang Jiang, Yijun Xu, Qi Zhu, Yingxia Wu, Zhe Wang, Shuang He, Shiyong Yu, Honggang Xiang

**Affiliations:** Department of General Surgery, Shanghai Pudong New Area People’s Hospital, Shanghai, 201299, China

**Keywords:** Colorectal cancer (CRC), epithelial-mesenchymal transition (EMT), myosin heavy chain 11 (*MYH11*), zinc finger E-box binding homeobox 1 (*ZEB1*), tumor progression

## Abstract

**Background:**

Colorectal cancer (CRC) is common and deadly, often leading to metastasis, challenging treatment, and poor outcomes. Understanding its molecular basis is crucial for developing effective therapies.

**Aims:**

This study aimed to investigate the role of Myosin Heavy Chain 11 (MYH11) in CRC progression, especially its effects on epithelial-mesenchymal transition (EMT) and cell behavior, and to explore its potential regulation by the EMT transcription factor zinc finger E-box binding homeobox 1 (ZEB1).

**Methods:**

Differential expression analysis was performed in the GSE123390 and TCGA-READ datasets, and 317 intersection genes were identified. The hub gene MYH11 was identified based on Protein-protein interaction (PPI) analysis and expression validation. The effects of MYH11 and the EMT transcription factor (ZEB1) on the behavior of CRC cells were investigated *in vitro*.

**Results:**

Bioinformatics research revealed that MYH11 was considerably downregulated in CRC samples as compared to normal samples. Overexpression of MYH11 inhibited the proliferation, migration, and invasion of CRC cells. Western blotting (WB) testing showed that MYH11 overexpression inhibited EMT by elevating E-cadherin levels while suppressing ZEB1, vimentin, and N-cadherin expressions. By contrast, overexpression of ZEB1 promoted EMT and enhanced migration, invasion, and proliferation of CRC cells. The negative impacts of MYH11 affecting EMT markers and cell behaviors were partially mitigated by co-overexpression of MYH11 and ZEB1, indicating that MYH11 regulates EMT and CRC progression through ZEB1.

**Conclusion:**

Our study shows MYH11 curbs CRC growth by blocking EMT and invasion, but ZEB1 overexpression reduces this effect. It uncovers key CRC pathways and suggests MYH11’s therapeutic potential.

## 1 Introduction

A tumor of malignant origin of the digestive system that develops inside the mucosal epithelium of the colon or rectum is known as colorectal cancer (CRC) [1]. According to the global cancer statistics in 2022, CRC is the third most common cancer worldwide, ranking third (after breast cancer and lung cancer). There were approximately 1.93 million new cases, with an age-standardized incidence rate (ASR) of 18.4 per 100,000 individuals [2]. In China, there were 517,106 new cases of CRC in 2022, with an ASR of 20.1 per 100,000, accounting for 12.2% of all cancer cases [3]. The etiology of CRC is multifaceted, and the incidence of CRC in China has increased markedly due to shifts toward Westernized dietary patterns, characterized by high consumption of processed meats, refined carbohydrates, and saturated fats, coupled with reduced intake of dietary fiber, fruits, and vegetables. These nutritional changes, combined with an aging population and sedentary lifestyles, have been strongly associated with elevated CRC risk in epidemiological studies [4,5]. In recent years, advances in genomic research have elucidated various molecular changes related to CRC and revealed key insights into its development and progression. For example, it was recently demonstrated that targeting MAPK interacting serine/threonine kinase 1 (*MKNK1*) affects the proliferation and metastasis of CRC cells, while also influencing their radioresistance [6]. It has already been shown that ubiquitin C-terminal hydrolase L3 (*UCHL3*) promotes the growth of CRC cells by controlling SRY-box transcription factor 12 (*SOX12*) through the AKT/mTOR signaling pathway [7]. The protein IGF2BP2 has been demonstrated to promote the development of CRC by upregulating the expression of the iron metabolism-related gene TFRC [8]. Moreover, the amplification of solute carrier family 12, member 5 (*SLC12A5*) has been observed in CRC, where it promotes tumor progression [9]. These findings underscore the significance of molecular mechanisms in CRC. As the field of CRC research continues to evolve, there is a pressing need for the development of novel diagnostic markers and more efficacious treatments. This underscores the imperative for further investigation into key oncogenes.

Myosin heavy chain 11 (*MYH11*) is a crucial component of smooth muscle contraction, playing a pivotal role in cellular motility and tissue homeostasis [10,11]. Emerging evidence indicates that dysregulation of *MYH11* is closely associated with tumor progression. For instance, in Gastric and CRC, *MYH11* expression is significantly downregulated, and mutations (e.g., missense mutations or deletions) have been linked to enhanced tumor invasiveness [12]. Additionally, *MYH11* is also closely associated with aortic aneurysms, breast cancer, and prostate cancer [13,14]. Recent studies have shown that *MYH11*+ fibroblasts (CAFs) play an important role in CRC, particularly in left-sided colorectal cancer (L-CRC), where they are more abundant and associated with strong tumor invasiveness and poor prognosis. These *MYH11*+ CAFs promote tumor cell migration and invasion through interactions with immune cells such as macrophages [15]. Additionally, *MYH11*+ CAFs have been found to be associated with tumor progression in lung adenocarcinoma (LUAD), suggesting similar mechanisms across different tumor types [16]. This indicates that *MYH11*+ CAFs may promote tumor progression by regulating key biological processes such as cell proliferation, invasion, and metastasis. A key process in cancer development, epithelial-mesenchymal transition (EMT), allows cells to take on mesenchymal characteristics, which improves their capacity for invasion and migration [17]. In CRC, the EMT plays a crucial role in tumor development and metastasis. For example, downregulation of pleckstrin homology-like domain family A member 2 (*PHLDA2*) can inhibit EMT in CRC across the PI3K/AKT signaling pathway, thereby hindering CRC progression [18]. *ZEB1* is an essential regulator of EMT, functioning as a transcription factor that represses epithelial markers while promoting mesenchymal genes [19]. It was previously demonstrated that *ZEB1* suppresses the transcriptional expression of epithelial genes, including E-cadherin, in cancer. In CRC, *ZEB1* is elevated and related to late-stage cancer metastasis and a poor prognosis [20]. The study by Sun et al. has shown that ubiquitin specific peptidase 10 (*USP10*) inhibits *ZEB1*-mediated CRC metastasis via controlling ZEB1 ubiquitination and stability of proteins [21]. Cai et al. found that the Transcriptional Repressor GATA Binding 1 (*TRPS1*) R544Q mutation significantly increased the invasion and migration of CRC *in vitro* and *in vivo* by regulating *ZEB1* [22]. Moreover, research has demonstrated that cAMP responsive element binding protein 1 (*CREB1*) functions as a transcriptional activator of *ZEB1*, and that *ZEB1*, a pivotal regulator of EMT, significantly enhances the migratory capacity and EMT process in colorectal cancer cells [23]. Despite these findings, the detailed mechanisms by which *ZEB1* regulates EMT in CRC remain incompletely understood. In particular, the upstream regulators of *ZEB1* and their interactions with downstream signaling pathways have not been fully elucidated. Based on these studies, understanding the interaction between *MYH11* and *ZEB1* and their role in tumor biology may provide insights into new therapeutic targets and prognostic markers.

The tumor microenvironment (TME), comprising stromal cells, immune cells, extracellular matrix (ECM) components, and signaling molecules, plays a pivotal role in cancer progression and metastasis [24]. In CRC, dynamic interactions between tumor cells and the TME facilitate processes such as immune evasion, angiogenesis, and EMT [25]. For instance, tumor-associated fibroblasts (CAFs) and macrophages within the TME secrete growth factors and cytokines that promote tumor cell invasion and chemoresistance [15,26]. Additionally, ECM remodeling driven by matrix metalloproteinases (MMPs) and integrins enhances metastatic potential. Emerging evidence suggests that cytoskeletal proteins, including myosin family members like MYH11, may influence TME dynamics by modulating cell-cell adhesion, mechanotransduction, and stromal crosstalk [13,27–29]. Given *MYH11*’s established role in smooth muscle contraction and cellular motility, its downregulation in CRC could disrupt tissue architecture, alter stromal signaling, and foster a permissive microenvironment for tumor progression. However, the precise mechanisms linking *MYH11* to TME regulation remain underexplored, warranting further investigation.

In CRC, EMT is critical for tumor development and metastasis. *ZEB1* is a major EMT transcription factor that enhances the motility and invasion of CRC cells according to previous studies. However, *MYH11* is a possible tumor suppressor, and its involvement in controlling EMT and CRC development is unknown [30]. In this research, we wanted to look into the impact of *MYH11* on CRC cell behavior and its regulatory interaction with *ZEB1*. By investigating the relationship between *MYH11* and *ZEB1*, we can get new insights into the molecular processes behind CRC development and novel treatment targets for metastasis reduction.

## 2 Material and Methods

### 2.1 Data Acquisition and Analysis of Differential Gene Expression

We retrieved the GSE123390 dataset from the GEO website (https://www.ncbi.nlm.nih.gov/gds/?term=GSE123390 (accessed on 01 June 2025)), which contains 28 CRC samples and 5 normal rectal tissue samples. 165 rectum adenocarcinoma (READ) samples and 10 normal samples comprised the TCGA-READ dataset, which is provided from the TCGA (https://www.cancer.gov/ccg/research/genome-sequencing/tcga (accessed on 03 June 2025)) website. The limma package in R programming (version 4.0) was utilized for recognizing differentially expressed genes (DEGs). Genes with fold change (FC) > 1.5 were categorized as up-regulated, while being down-regulated if FCp < 0.67 [31]. These thresholds are commonly used in the field of gene expression analysis to identify significant changes in gene expression levels. A difference was considered significant if *p* was less than 0.05. To visualize the DEGs, the ggplot2 software in R was utilized.

### 2.2 Consensus Clustering Analysis of GSE123390-DEGs

To explore potential molecular subtypes of CRC, unsupervised consensus clustering was performed based on the expression profiles of 193 upregulated and 248 downregulated DEGs identified from the GSE123390 dataset. The analysis was conducted using the “ConsensusClusterPlus” R package (version 1.54.0) with 1000 repetitions to ensure clustering stability. The parameters were set as follows: maximum number of clusters (maxK) = 6, resampling ratio = 0.8, clustering algorithm = partitioning around medoids (PAM), and distance metric = “1 − Pearson correlation”. The optimal number of clusters (k) was determined by evaluating the cumulative distribution function (CDF) curve, delta area plot, and consensus matrix heatmap. Gene expression patterns of the identified clusters were visualized using hierarchical clustering and heatmaps generated with the “pheatmap” R package (version 1.0.12).

### 2.3 Identification and Functional Enrichment Analysis of Intersection DEGs

The intersection of DEGs of TCGA-READ and GSE123390 datasets was evaluated via the Bioinformatics and Evolutionary Genomics (http://bioinformatics.psb.ugent.be/webtools/Venn/(accessed on 01 June 2025)) database. The DAVID (https://david.ncifcrf.gov) database was then utilized to analyze the resultant intersection DEGs for gene ontology (GO) and Kyoto Encyclopedia of Genes and Genomes (KEGG) enrichment.

### 2.4 Protein-Protein Interaction (PPI) Network and Key Intersection Gene Expression Analysis

The obtained intersection genes were analyzed by PPI network analysis using the STRING (https://string-db.org/) website. The top ten genes were identified by maximum clique centrality (MCC) and maximum neighborhood component (MNC) algorithms using the Cytohubba plugin (version 3.2.0) in Cytoscape. The top ten genes from the two methods were then cross-analyzed using the Bioinformatics and Evolutionary Genomics website to identify significant interaction genes. The expression of the obtained key intersection genes in normal and tumor samples of the TCGA-READ and GSE123390 datasets was analyzed using the SangerBox (http://sangerbox.com/) website. For *p* < 0.05, the results were meaningful.

### 2.5 Cell Culture

The ATCC (American Type Culture Collection, Manassas, VA, USA) provided human CRC cell lines RKO, HT29, HCT-15, and HCT116, along with human normal colon fibroblast cell line CCD-18Co. All cell lines were cultured using DMEM (11965092, Invitrogen, Carlsbad, CA, USA) supplemented with 10% FBS (A5669701, Thermo Fisher scientific, Waltham, MA, USA) and 1% penicillin-streptomycin (C0222, Beyotime, Shanghai, China) was utilized to cultivate the cells. All cell lines were cultured at 37°C with 5% CO_2_ in a humidified incubator and tested for mycoplasma contamination, with negative results.

### 2.6 Cell Transfection

To achieve overexpression of *MYH11* and *ZEB1*, HT29 and HCT116 cells were transfected using 10 µL Lipofectamine^TM^ 2000 reagent (11668019, Invitrogen) following the directions provided by the manufacturer. Plasmids pcDNA-3.1-GFP encoding *MYH11* (pcDNA-3.1-GFP-MYH11) or *ZEB1* (pcDNA-3.1-GFP-ZEB1) were utilized to transfect cells, while an empty vector served as a control. These plasmids were obtained from Invitrogen (V79020, Invitrogen). After 48 h of incubation, transfection efficiency was assessed by observing green fluorescence under a fluorescence microscope (Olympus BX 60 fluorescence microscope, Japan), and the cells were collected for subsequent experiments. During the initial stages of our experiments, we compared the expression levels between the control group and the vector group. No significant differences were observed between these two groups. Therefore, we used the vector group as the sole control for subsequent experiments.

Transfect CRC cells with specific small interfering RNA (siRNA) targeting *MYH11* or *ZEB1*, designed and synthesized by Shanghai GenePharma, to achieve knockdown of *MYH11* or *ZEB1* expression. siRNA sequences for *MYH11*, *ZEB1* and si-NC are as follows: si-*MYH11*-1: Sense: 5^′^-CAG AUGAUGAGAUGUUCCAGGAAA-3^′^, Anti-sense: 5^′^-UUUCCUGGAACAUCUCAUCAUCCUG-3^′^; si-*MYH11*-2: Sense: 5^′^-CAGGCUCAGAUGAAGGACUUUCAAA-3^′^, Anti-sense: 5^′^-UUUGAAAGUCCUUCAUCUGAGCCUG-3^′^; si-*ZEB1*: Sense: 5^′^-CGAGAGAGAGAGUUUGACAAGGGAA-3^′^, Anti-sense: 5^′^-UUCCCUUGUCAAACUCUCUCUCUCG-3^′^; si-NC (negative control): Sense: 5^′^-CGAAGAGUGAGAGUUGAACGAGGAA-3^′^, Anti-sense: 5^′^-UUCCUCGUUCAACUCUCACUCUUCG-3^′^. Following the manufacturer’s instructions, cells were transfected using Lipofectamine 2000^TM^ (11668019, Invitrogen).

### 2.7 Western Blotting (WB) Assay

Proteins were extracted from cells using RIPA buffer (G2002, Servicebio, Wuhan, China) augmented with protease inhibitors (P1005, Beyotime, Shanghai, China), and protein concentrations were measured using the BCA protein assay kit (PC0020, Solarbio, Beijing, China). Equal amounts of protein were separated by SDS-PAGE and transferred to PVDF (Polyvinylidene fluoride) membranes (YA1701, Solarbio). Membranes were blocked with 5% non-fat milk in TBST (G0004, Servicebio, Wuhan, China) for 1 h at room temperature, followed by incubation with primary antibodies overnight at 4°C. After washing, membranes were incubated with appropriate HRP-conjugated secondary antibodies (D110157, 1:5000, Sangon, Shanghai, China) for an hour at room temperature. The ECL detection system was utilized to observe the protein bands, and ImageJ software (version 2.0.0, https://imagej.net/) was utilized to quantify them. Following are the primary antibodies employed in this investigation: MYH11 (ab133567, 1:1000, Abcam, Cambridge, UK), N-cadherin (ab245117, 1:5000, Abcam), E-cadherin (ab314063, 1:1000, Abcam), ZEB1(ab203829, 1:1000, Abcam), Vimentin (ab92547, 1:1000, Abcam), GAPDH (ab181602, 1:2500, Abcam).

### 2.8 Quantitative Real-Time PCR (qRT-PCR)

The TRIzol reagent (R110, Solarbio) was utilized for obtaining total RNA from CRC cells. Complementary DNA (cDNA) synthesis was conducted utilizing the PrimeScrip™ RT reagent kit (RR047A, Takara, Kameoka, Kyoto Prefecture, Japan). qRT-PCR was conducted utilizing a Bio-Rad apparatus (Bio-Rad, Hercules, CA, USA) and One Step TB Green® PrimeScript™ RT-PCR Kit (RR066A, Takara) according to standard amplification conditions. *GAPDH* was employed as a standard to assess the expression levels of the genes to be evaluated, and the 2^−ΔΔCt^ technique was utilized to determine the relative expression [32]. The following primers were specifically utilized in this experiment: *MYH11* (forward: 5^′^-GTATCACGCAAGGCCCATCT-3^′^, reverse: 5^′^-ATGTCCTCTCGTCTCTGGCT-3^′^); *ZEB1* (forward: 5^′^-GCTGTTTCAAGATGTTTCCTTCCA-3^′^, reverse: 5^′^-GCCTATGCTCCACTCCTTGC-3^′^); *GAPDH* (forward: 5^′^-TGTTGCAACCGGGAAGGAAA-3^′^, reverse: 5^′^-GCATCACCCGGAGGAGAAAT-3^′^). All primers used for gene amplification in this study were designed specifically for the human species.

### 2.9 Cell Proliferation Assays

Cell counting kit-8 (CCK-8; C0037, Beyotime) test was utilized to evaluate cell viability in CRC cell lines. The CCK-8 assay is a widely used and reliable method for measuring cell viability. It is based on the reduction of the water-soluble tetrazolium salt WST-8 to a soluble formazan dye by dehydrogenases in living cells. This assay is characterized by its simplicity, sensitivity, and non-toxicity to cells. After being planted at a density of 5 × 10^3^ cells per well in 96-well plates, the cells were left to adhere for the whole night. At days 0, 1, 3, 5, and 7, 10 µL of CCK-8 solution was applied to each well in accordance with the specified treatment protocols. The cells were then incubated for an extra two hours at 37°C. A microplate reader (Bio-Rad, Model 680, USA) was utilized to measure the absorbance at 450 nm.

For the colony formation assay, cells were seeded in 6-well plates at a density of 500 cells per well and cultured for 14 days until visible colonies formed. Following the incubation period, cells were treated with 4% paraformaldehyde (P1110, Solarbio) for 15 min. Subsequently, colonies were stained with nitro blue tetrazolium chloride (NBT; ST363, Beyotime). Excess dye was removed by rinsing with PBS, and colonies were tallied utilizing a light microscope (CX23 model, Olympus Corporation).

### 2.10 Transwell Assay

Transwell experiments were carried out to assess the migratory and invasive capabilities of CRC cells. A Transwell insert (140629, Invitrogen) had its top chambers pre-coated with Matrigel (M8370, Solarbio) for the invasion test. In the upper chamber, cells were planted at a density of 1 × 10^5^ cells after being placed in serum-free DMEM. 10% FBS was added to DMEM, which was used to fill the lower chamber. After a whole day of incubation at 37°C, the migrated cells on the bottom surface were fixed with 4% paraformaldehyde for 15 min, and the non-migrated cells on the top surface were removed with a cotton swab. After 10 min of labeling with 1 μg/mL DAPI (C1006, Beyotime), the cells were counted utilizing a fluorescence microscope (Olympus BX 60 fluorescence microscope, Japan). For the migration test, the upper chamber was not pretreated with Matrigel, and the rest of the operations were consistent with the invasion assay.

### 2.11 Statistical Analysis

R programming was employed for all statistical analyses to evaluate the significance of the experimental results in this study. The mean ± standard deviation (SD) of the data was displayed. Unpaired Student’s *t*-tests were utilized for comparisons between two groups, while one-way analysis of variance followed by Tukey’s post hoc test was used for multiple group comparisons. *p-*value less than 0.05 was the threshold for statistical significance. To guarantee repeatability, every experiment was conducted at least three times.

## 3 Results

### 3.1 Screening and Enrichment Analysis of DEGs

**Figure 1:**
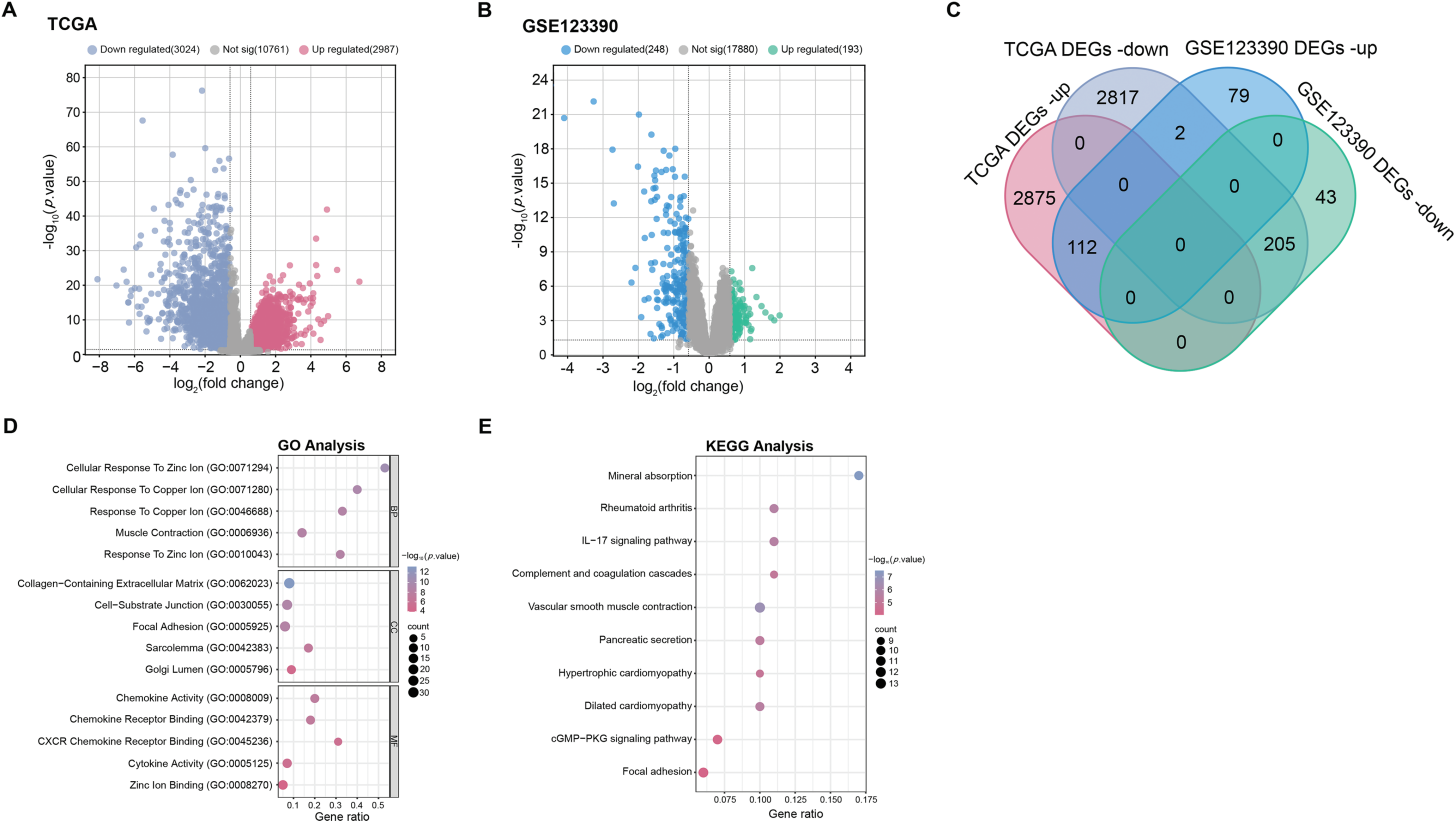
Screening of DEGs and enrichment analysis. (**A**,**B**) The volcano plots of DEGs in TCGA-READ (**A**) and GSE123390 (**B**) datasets. Each point on the graph corresponds to a gene. The *x*-axis (log_2_(fold change)) represents the log fold change in gene expression between tumor and normal samples. The *y*-axis (−log_10_(*p*-value)) represents the statistical significance of the observed differential expression, with higher values indicating greater significance. (**C**) Venn diagram of the intersection analysis of up-regulated and down-regulated DEGs in TCGA-READ and GSE123390 datasets. The overlapping parts represent intersection genes. (**D**) GO analysis shows enriched biological processes, cellular components, and molecular functions associated with DEGs. The *x*-axis represents the gene ratio, and the *y*-axis lists the GO terms. The size of the dots indicates the number of genes involved in each term. (**E**) KEGG enrichment analysis predicts the pathways involved in the intersection of genes. The horizontal axis represents the gene ratio, and the vertical axis represents the enrichment term. TCGA-READ: The cancer genome atlas-rectum adenocarcinoma, DEGs: Differentially expressed genes, KEGG: Kyoto encyclopedia of genes and genomes, GO: Gene ontology, BP: Biological processes, CC: Cellular components, MF: Molecular functions

Differential expression analysis was performed on the TCGA-READ and GSE123390 datasets. The results illustrated that 3024 down-regulated DEGs and 2987 up-regulated DEGs were screened in the TCGA-READ dataset (Fig. 1A), while 248 down-regulated DEGs and 193 up-regulated DEGs were screened in the GSE123390 dataset (Fig. 1B). A total of 317 intersecting genes were found by intersection analysis of these up-regulated and down-regulated DEGs (Fig. 1C). Subsequently, functional enrichment analysis was performed on these 317 intersection genes, showing significant GO enrichment. In terms of biological process (BP), these genes were significantly enriched in response to copper ion, cellular response to copper ion, and cellular response to zinc ion. On cellular component (CC), these genes were markedly enriched in the focal adhesion, cell-substrate junction, and collagen-containing extracellular matrix. Regarding molecular function (MF), these genes were considerably enriched in CXCR chemokine receptor binding, chemokine receptor binding, and chemokine activity (Fig. 1D). Genes were substantially enriched in pathways such as the IL-17 signaling pathway, dilated cardiomyopathy, and cGMP-PKG signaling pathway according to the analysis of KEGG pathways (Fig. 1E).

### 3.2 Consensus Clustering Reveals Five Distinct Molecular Subtypes in CRC

To explore the molecular heterogeneity between CRC samples, we performed consensus clustering analysis on differentially expressed genes in the GSE123390 dataset. The CDF plot showed that the higher the k value, the higher the cluster stability, and the smoother and more saturated the curve (Supplementary Fig. S1A). To determine the optimal number of clusters, we examined the relative change in the area under the CDF curve (incremental area). As shown in Supplementary Fig. S1B, the maximum increase in area occurred between k = 2 and k = 3, while the gain gradually decreased when k > 3. Notably, the incremental area reached a stable state at k = 5, indicating that dividing the samples into five clusters can achieve a balance between stability and resolution. The consensus matrix heat map of k = 5 results showed that each of the five identified molecular subtypes (C1 to C5) showed a different clustering pattern (Supplementary Fig. S1C). Hierarchical clustering was then used to visualize the expression profiles of all DEGs in the five clusters. As shown in the heat map, the gene expression patterns varied greatly between the subtypes, and each cluster showed a unique transcriptional signature (Supplementary Fig. S1D). These results highlight the molecular heterogeneity within CRC and suggest that the identified subtypes may have different biological characteristics and clinical significance.

### 3.3 PPI Network and Gene Expression Analysis to Identify Hub Gene

**Figure 2:**
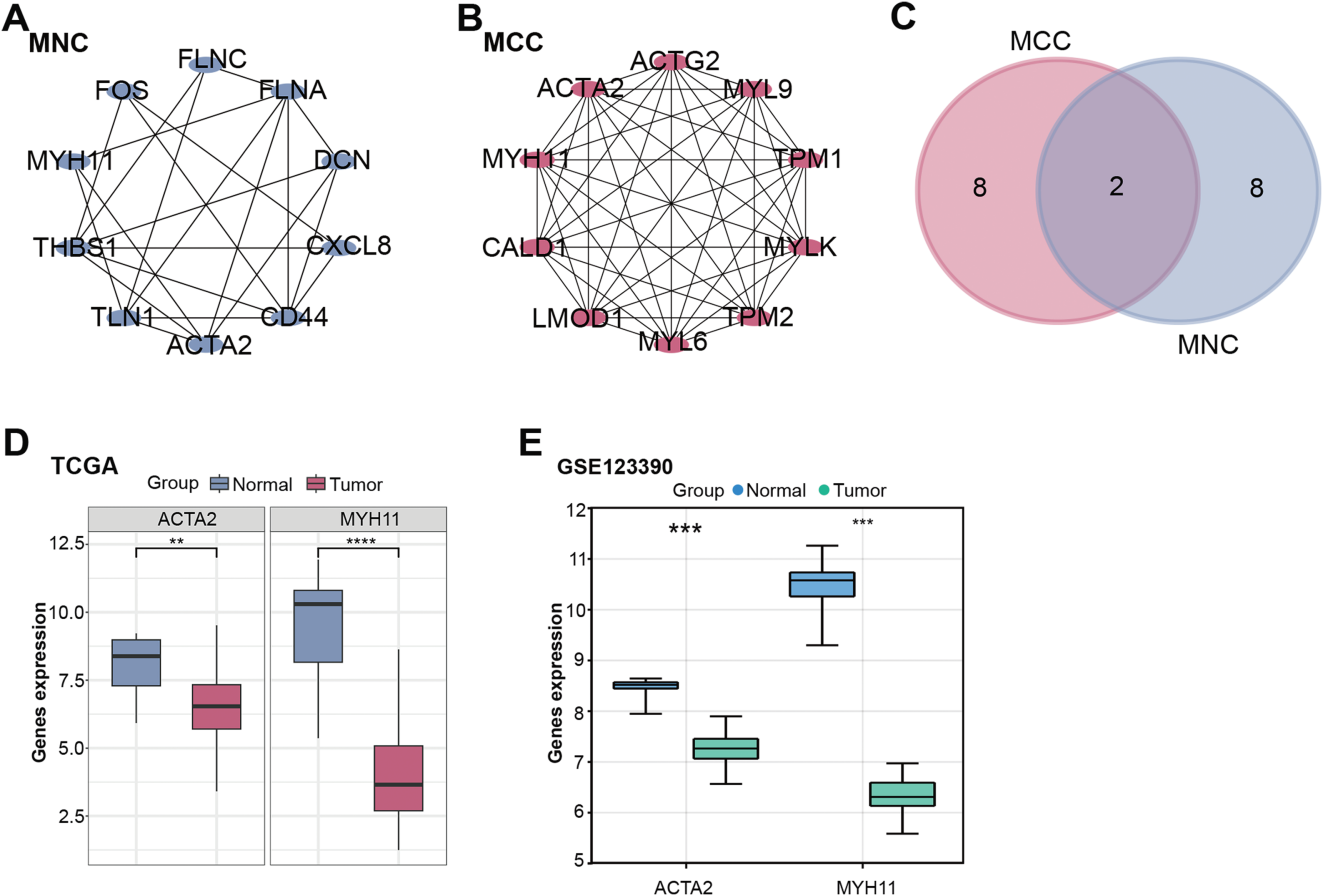
PPI network and gene expression analysis obtained a hub gene. (**A**,**B**) The MNC (**A**) and MCC (**B**) algorithms were used to identify the top ten genes for PPI network analysis. Each point represents the top ten genes ranked by the algorithm, and each line represents the association between genes. (**C**) Venn diagram of the intersection analysis of the top ten genes of the MNC and MCC algorithms. The overlapping part in the middle is the key intersection gene screened out. (**D**,**E**) Box plots of gene expression of key intersection genes (*ACTA2* and *MYH11*) in normal and tumor samples from the TCGA-READ (**D**) and GSE123390 (**E**) datasets. **p < 0.01, ***p < 0.001, ****p < 0.0001. PPI: Protein-protein interaction, MNC: Maximum neighborhood component, MCC: Maximum correlation criterion, TCGA-READ: The cancer genome atlas-rectum adenocarcinoma

PPI network analysis further explored these intersection genes. MNC and MCC algorithms show the top 10 genes. Among them, 10 nodes and 22 edges compose the MNC network (Fig. 2A). The MCC network consists of 10 nodes and 45 edges (Fig. 2B). Afterwards, intersection analysis was also performed on the top 10 genes of the MNC and MCC algorithms, and two key intersection genes, actin alpha 2, smooth muscle (*ACTA2*) and *MYH11*, were obtained (Fig. 2C). The expression of these two key intersection genes was analyzed in the normal group and tumor group of TCGA-READ and GSE123390 datasets. The findings indicated that the expression of *ACTA2* [33,34] and *MYH11* in tumor samples of both datasets was significantly lower than that in normal samples, and the low expression of *MYH11* in tumor samples was more significant (Fig. 2D,E). Therefore, *MYH11* was assigned as the hub gene for this investigation.

### 3.4 Overexpression of MYH11 Inhibits CRC Cell Proliferation

**Figure 3:**
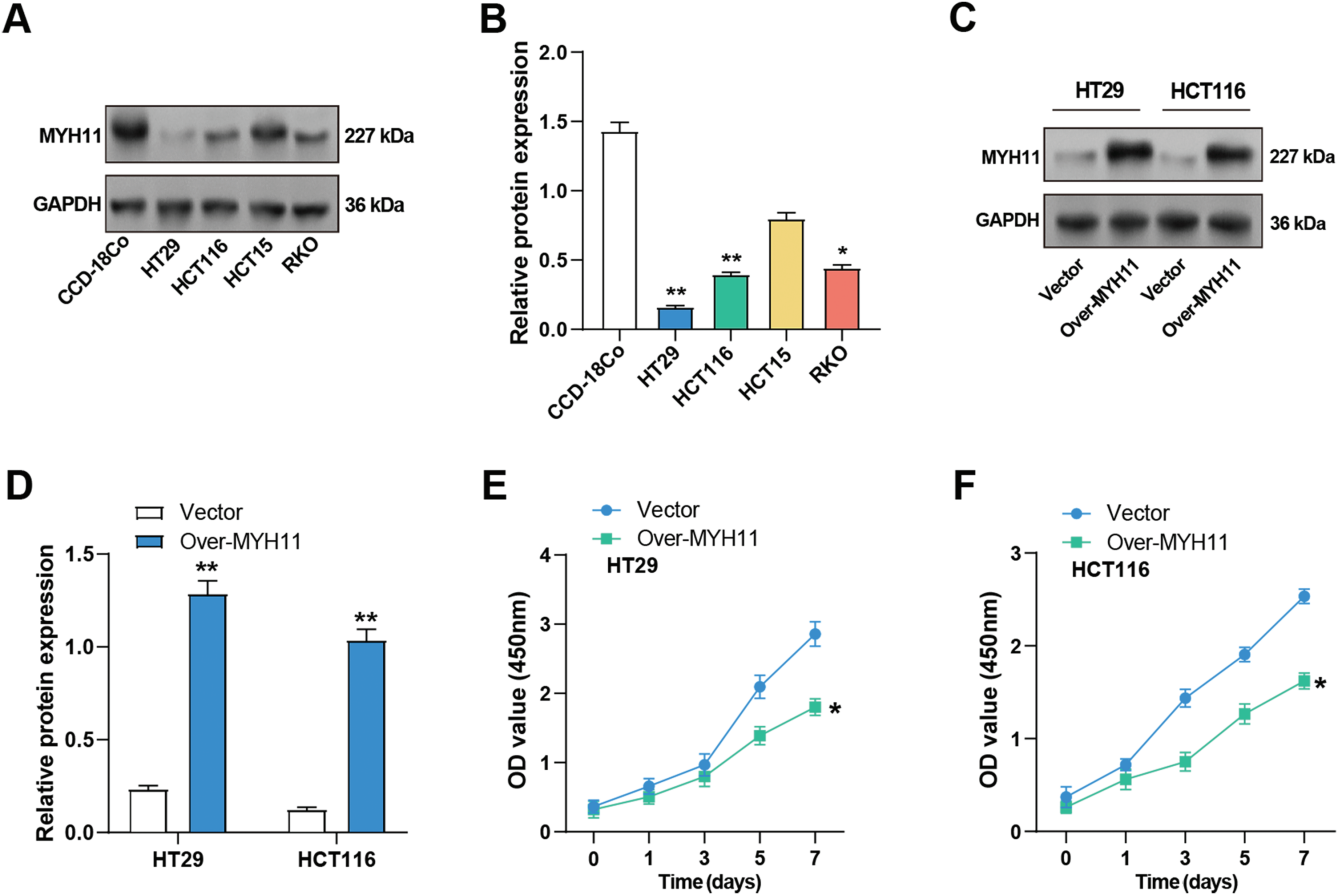
Overexpression of *MYH11* inhibits the proliferation of CRC cells. (**A**,**B**) WB detected the protein expression level of MYH11 in CRC cells (HT29, HCT116, HCT15, and RKO) and CCD-18Co cells, and performed quantitative analysis. (**C**,**D**) WB detected the protein expression level of MYH11 in CRC cells overexpressing MYH11 and performed a quantitative analysis. (**E**,**F**) CCK-8 detected the cell viability of CRC cells overexpressing *MYH11*. The *x*-axis is time (0, 1, 3, 5, 7 days), and the *y*-axis is the OD value at 450 nm absorbance. *p < 0.05, **p < 0.01. CRC: Colorectal cancer, WB: Western blotting, CCK-8: Cell counting kit-8, OD: Optical density

MYH11 protein expression levels in CRC and CCD-18Co cells were determined by WB analysis. The results demonstrated a notable reduction in MYH11 expression levels in CRC cell lines relative to CCD-18Co cells, particularly in HCT116 and HT29 cells (Fig. 3A,B). Accordingly, these two cell lines were selected for the subsequent experiment. Given the pivotal role of EMT in cancer progression and the well-established function of ZEB1 as a key EMT regulator, we further explored the potential interplay between ZEB1 and MYH11 in CRC cells. Transfection efficiency of MYH11 and ZEB1 overexpression was evaluated by observing GFP fluorescence under a fluorescence microscope. Successful transfection and protein expression were confirmed by green fluorescence, with a transfection efficiency of over 50% (Supplementary Fig. S2A,B). Subsequently, the efficacy of MYH11 overexpression in HT29 and HCT116 cells was corroborated through WB analysis. The expression levels of MYH11 in both cell lines were markedly elevated following overexpression in comparison to the control group (Fig. 3C,D). The CCK-8 test was utilized to measure the effect of *MYH11* overexpression on cell viability over seven days. The findings indicated that, in contrast to the control group, *MYH11* overexpression markedly diminished the viability of CRC cells (Fig. 3E,F). These outcomes imply that *MYH11* overexpression impedes CRC cell proliferation.

### 3.5 MYH11 Overexpression Prevents the CRC Cells Growth In Vitro

**Figure 4:**
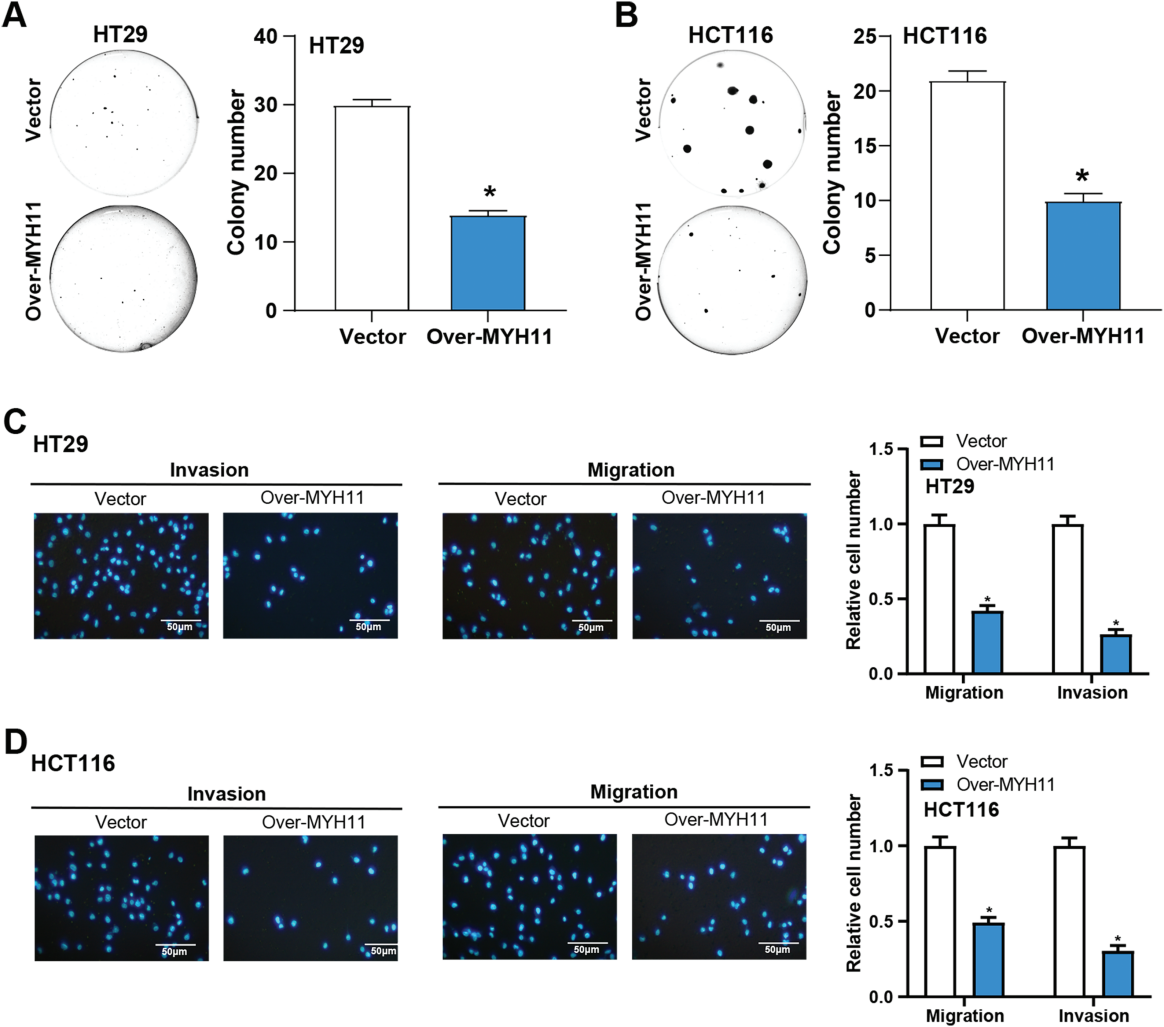
*MYH11* overexpression inhibits the growth of CRC cells *in vitro*. (**A**,**B**) Colony formation assay detected the colony formation ability of HT29 (**A**) and HCT116 (**B**) cells overexpressing *MYH11*, and quantitative analysis of colony numbers. (**C**,**D**) Transwell detected the invasion and migration abilities of HT29 (**C**) and HCT116 (**D**) cells overexpressing *MYH11*, and quantitative analysis. Scale bar: 50 μm. *p < 0.05. CRC: Colorectal cancer

The colony formation tests were first conducted to examine the effects of *MYH11* overexpression on CRC cell invasion and migration. The findings demonstrated that overexpression of *MYH11* dramatically reduced the colony-forming ability of CRC cells in comparison to control (Fig. 4A,B), highlighting its role in inhibiting cell proliferation. The invasion and migration assays were carried out to evaluate the effect of *MYH11* overexpression on these cellular behaviors. The results demonstrated that overexpressed *MYH11* markedly decreased both the migration and invasion capabilities of CRC cells (Fig. 4C,D). All of these findings collectively point to the inhibition of CRC cell invasion and migration by *MYH11* overexpression.

### 3.6 Overexpression of MYH11 Inhibits EMT of CRC Cells

**Figure 5:**
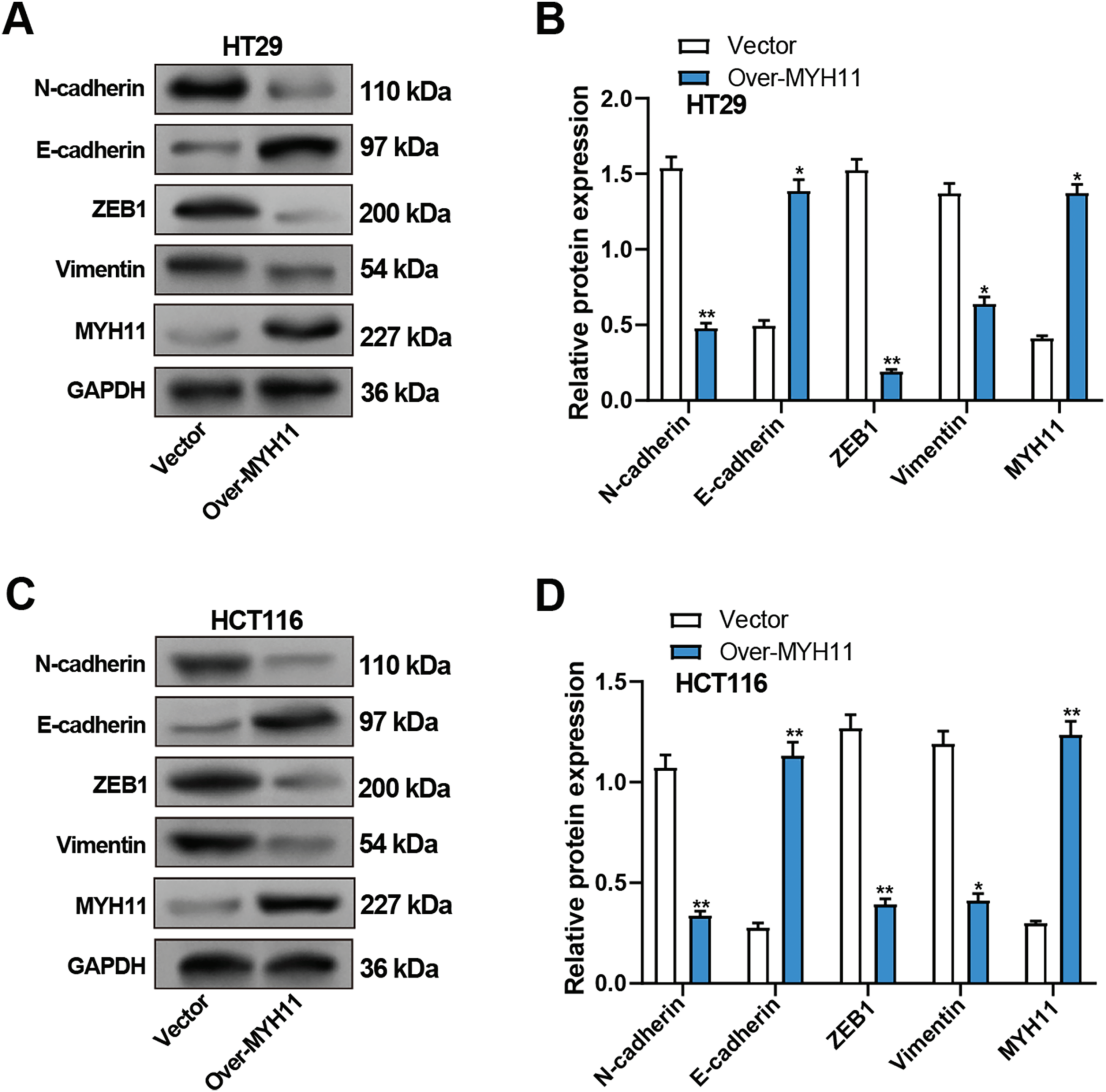
Overexpression of MYH11 inhibits EMT in CRC cells. (**A**,**B**) WB detected the protein expression levels of MYH11 and EMT-related proteins (E-cadherin, N-cadherin, Vimentin, and ZEB1) in HT29 cells overexpressing *MYH11*, with representative images shown in (**A**) and quantitative analysis in (**B**). (**C**,**D**) WB detected the protein expression levels of MYH11 and EMT-related proteins in HCT116 cells overexpressing MYH11, as illustrated by the representative blot images (**C**) and corresponding quantification (**D**). (**C**) Representative WB images; (**D**) Quantification of band intensities. *p < 0.05, **p < 0.01. CRC: Colorectal cancer, WB: Western blotting, EMT: Epithelial-mesenchymal transition

We detected the EMT-related proteins (Vimentin, N-cadherin, E-cadherin, ZEB1) expression in CRC cells by WB assay to explore the impact of MYH11 overexpression on EMT of CRC cells. Compared with the control group, the results revealed that overexpression of MYH11 significantly upregulated E-cadherin expression, whereas concurrently downregulating N-cadherin, Vimentin, and ZEB1 levels in CRC cells (Fig. 5A–D).

To further validate the impact of *MYH11* on EMT in CRC cells, we knocked down *MYH11* using siRNA. The knockdown efficiency was verified by qRT-PCR and WB (Supplementary Fig. S3A–C). The effects of *MYH11* knockdown on EMT-related proteins were assessed by WB. The results showed that *MYH11* knockdown significantly downregulated E-cadherin expression while upregulating the levels of N-cadherin, vimentin, and ZEB1 in CRC cells (Supplementary Fig. S3D–G).

### 3.7 Overexpression of ZEB1 Promotes EMT

**Figure 6:**
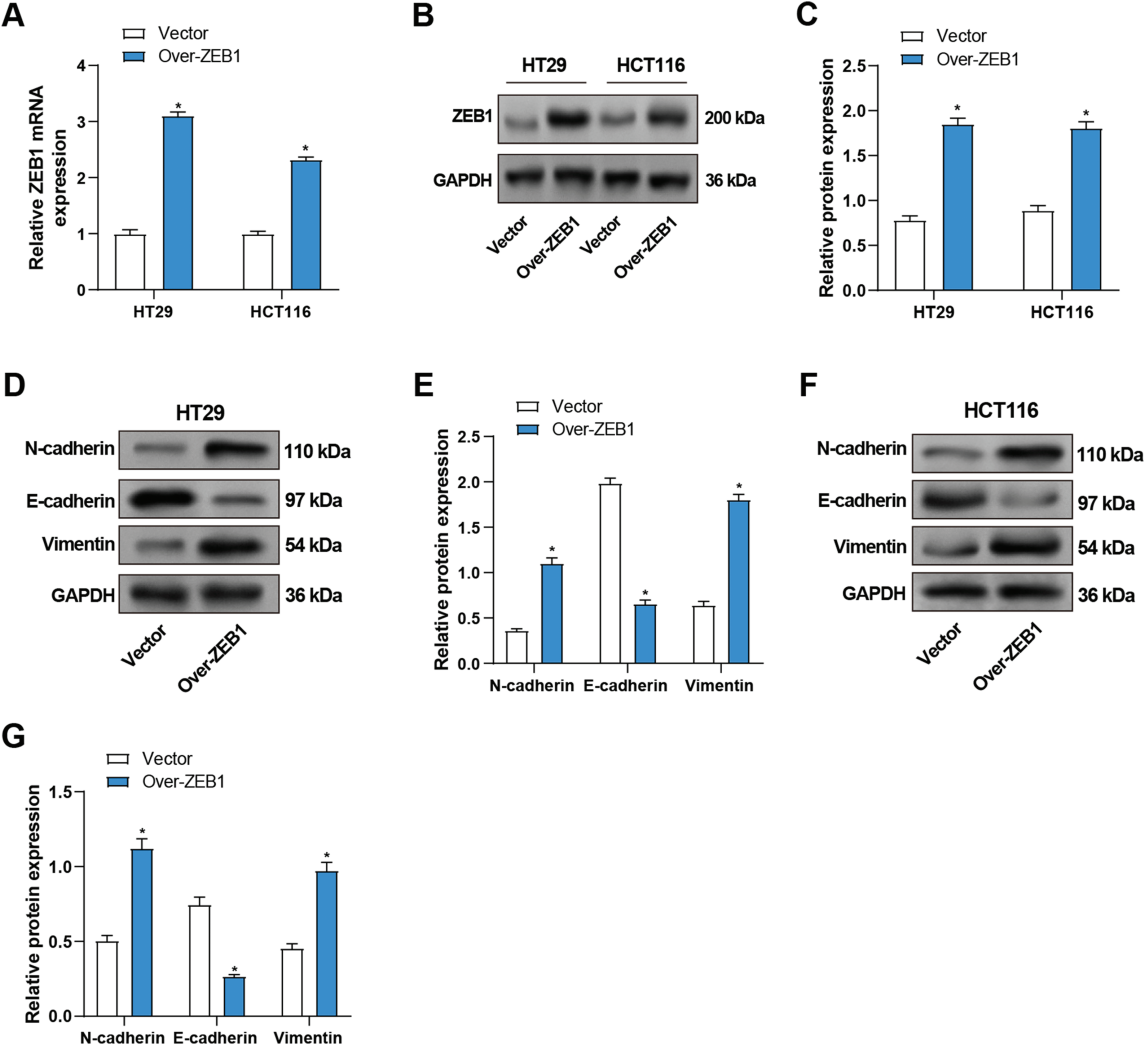
Overexpression of *ZEB1* promotes EMT in CRC cells. (**A**) qRT-PCR detected *ZEB1* mRNA expression in CRC cells overexpressing *ZEB1*. (**B**,**C**) WB detected the protein expression level of ZEB1 in CRC cells overexpressing *ZEB1*, and performed quantitative analysis. (**D**–**G**) WB detected the protein expression levels of EMT-related proteins (E-cadherin, N-cadherin, and Vimentin) in HT29 (**D**,**E**) and HCT116 (**F**,**G**) cells overexpressing *ZEB1*, and quantitative analysis. *p < 0.05. CRC: Colorectal cancer, qRT-PCR: Quantitative real-time PCR, WB: Western blotting, EMT: Epithelial-mesenchymal transition

To evaluate the efficiency of *ZEB1* overexpression in CRC cells, qRT-PCR and WB were utilized. *ZEB1*-overexpressing CRC cells showed considerably higher levels of *ZEB1* mRNA and protein expression than the control group (Fig. 6A–C). Furthermore, WB evaluated the expression of proteins linked to EMT. Overexpression of *ZEB1* was associated with significantly enhanced levels of mesenchymal markers, including vimentin and N-cadherin, while reducing the expression of E-cadherin (Fig. 6D–G). In conclusion, *ZEB1* overexpression promoted EMT in CRC cells and contributed to the metastasis of CRC.

### 3.8 Overexpression of ZEB1 Promotes the Cellular Behavior of CRC Cells In Vitro

**Figure 7:**
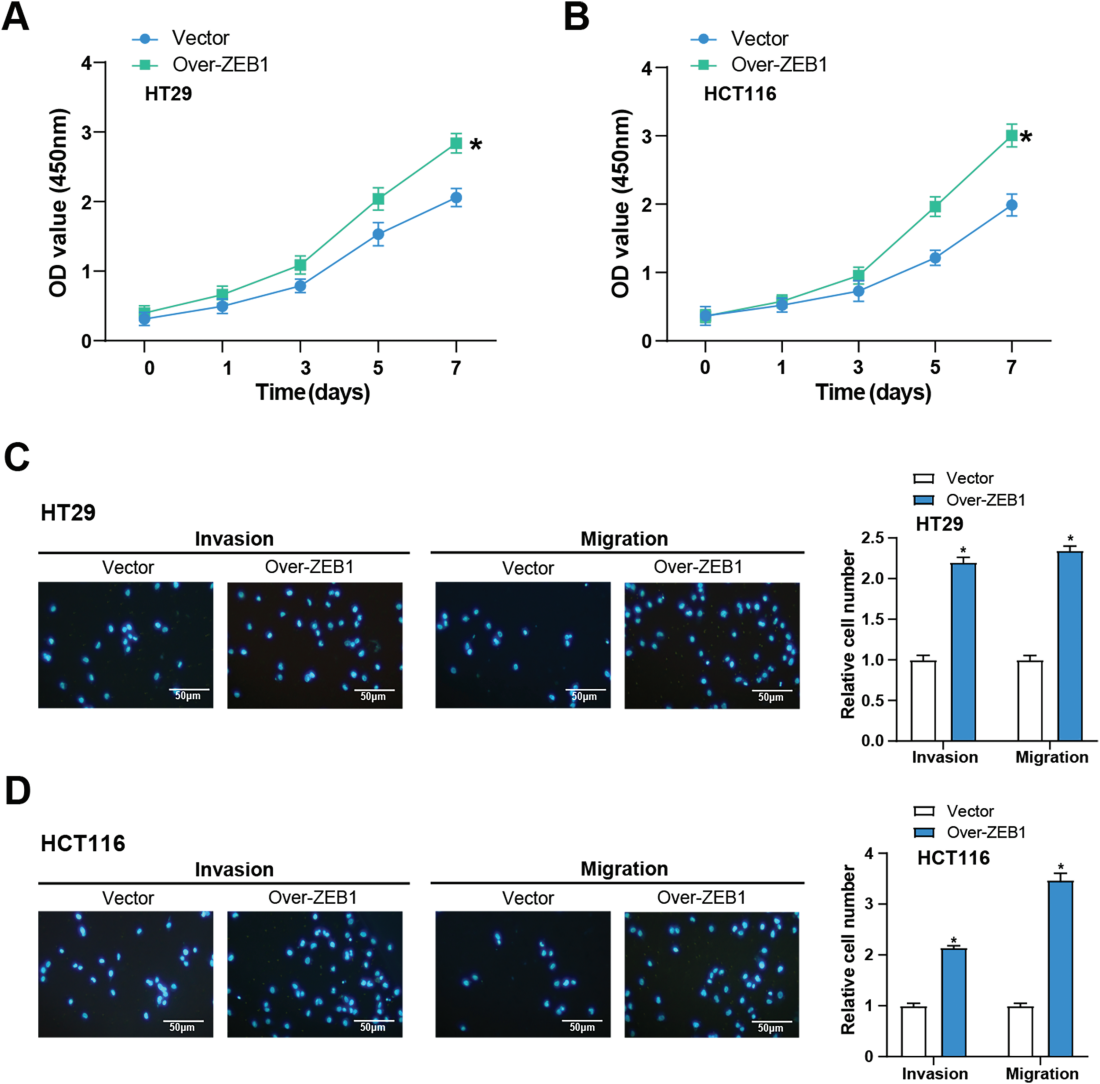
Overexpression of *ZEB1* promotes the cellular behavior of CRC cells. (**A**,**B**) CCK-8 detected the cell viability of CRC cells overexpressing *ZEB1* on 0, 1, 3, 5, and 7 days. The *x*-axis is time, and the *y*-axis is OD value at 450 nm absorbance. (**C**,**D**) Transwell detected the invasion and migration abilities of HT29 (**C**) and HCT116 (**D**) cells overexpressing *ZEB1*, and quantitative analysis. Scale bar: 50 μm. *p < 0.05. CRC: Colorectal cancer, CCK-8: Cell counting kit-8, OD: Optical density

We performed the following series of tests to learn more about the function of *ZEB1* in the cellular behavior of CRC cells. CCK-8 tests were utilized to evaluate the impact of *ZEB1* overexpression on CRC cell viability. In comparison to the control group, the results demonstrated a considerable increase in CRC cell viability following *ZEB1* overexpression (Fig. 7A,B). Additionally, transwell tests also served to assess the migratory and invasive capabilities of these cells. The findings showed that *ZEB1* overexpression markedly enhanced migration and invasion (Fig. 7C,D). These findings suggest that *ZEB1* plays a significant role in promoting aggressive tumor behavior.

### 3.9 MYH11 Regulates the EMT of CRC Cells by Targeting ZEB1

**Figure 8:**
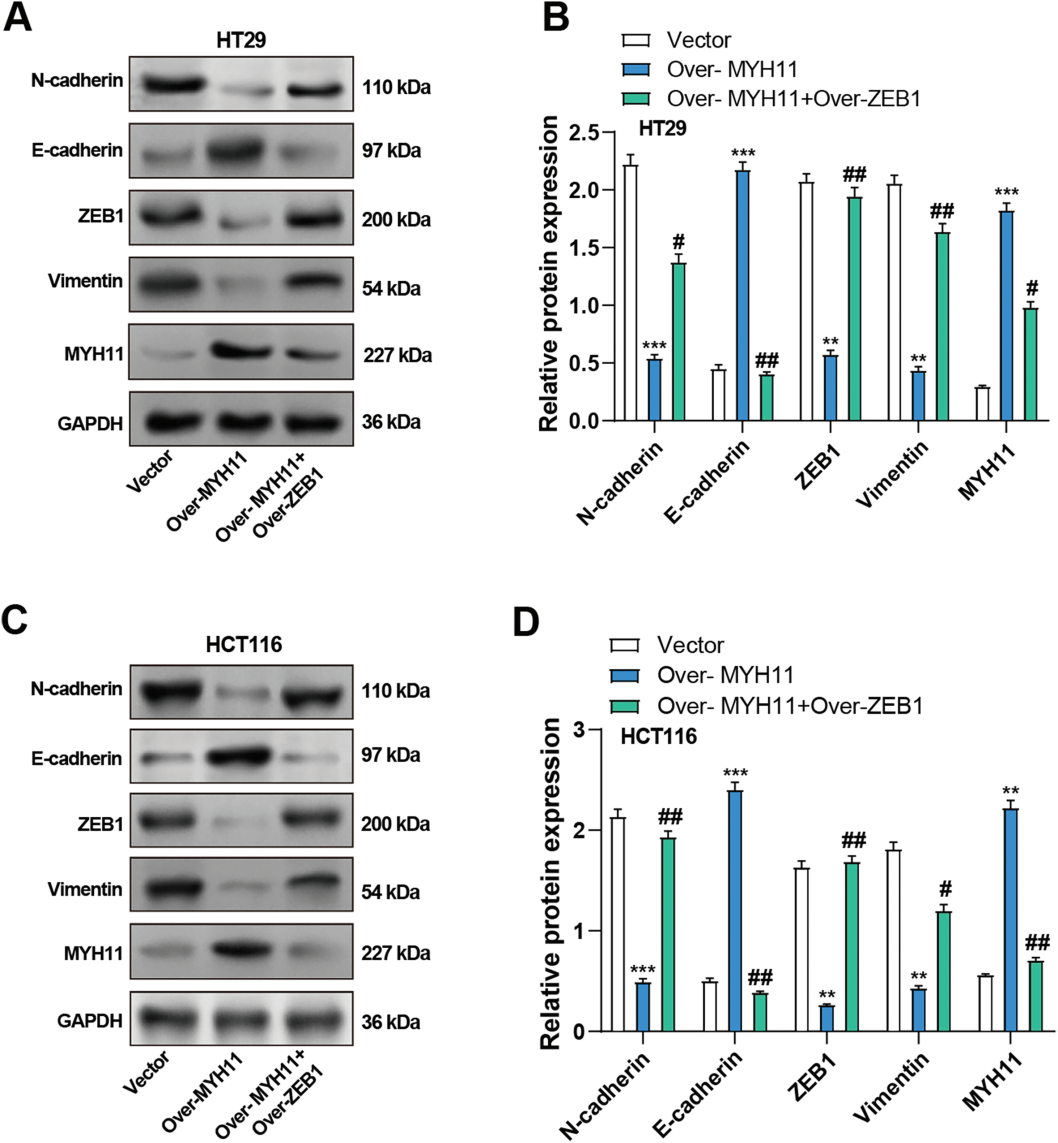
Co-overexpression of MYH11 and ZEB1 alters EMT-related protein expression in HT29 and HCT116 cells. (**A**) WB analysis of MYH11 and EMT markers (E-cadherin, N-cadherin, ZEB1, and Vimentin) in HT29 cells transfected with MYH11 alone or co-transfected with MYH11 and ZEB1. (**B**) Quantitative analysis of protein expression levels in HT29 cells. (**C**) Western blot analysis of EMT markers in HCT116 cells with the same transfection conditions. (**D**) Quantitative analysis of protein expression levels in HCT116 cells. **p < 0.01, ***p < 0.001 vs. Vector. ^#^*p* < 0.05, ^##^*p* < 0.01 vs. Over-MYH11. CRC: Colorectal cancer, WB: Western blotting, EMT: Epithelial-mesenchymal transition

The impacts of MYH11 and ZEB1 on the EMT of CRC cells were investigated using WB analysis to evaluate the potential mechanism of *MYH11*. The findings demonstrated that *MYH11* overexpression significantly increased the levels of E-cadherin, whereas it dramatically reduced the levels of ZEB1, N-cadherin, and vimentin compared with the control group. Notably, co-overexpression of *ZEB1* and *MYH11* partially restored the expression of mesenchymal markers and ZEB1, while considerably reversing the higher E-cadherin expression level relative to *MYH11* overexpression (Fig. 8A–D). Additionally, we performed *ZEB1* knockdown using siRNA and verified the knockdown efficiency by qRT-PCR and WB (Supplementary Fig. S4A−C). Notably, the co-knockdown of *ZEB1* and *MYH11* partially reversed the expression of mesenchymal markers and *ZEB1*, while correspondingly restoring the lower E-cadherin expression level relative to *MYH11* knockdown (Supplementary Fig. S4D−F). These findings suggest that *MYH11* influences EMT in CRC cells, at least in part, by targeting *ZEB1*, indicating their potential significance in tumor development and metastasis.

### 3.10 MYH11 Regulates the Cell Behavior of CRC Cells through ZEB1

**Figure 9:**
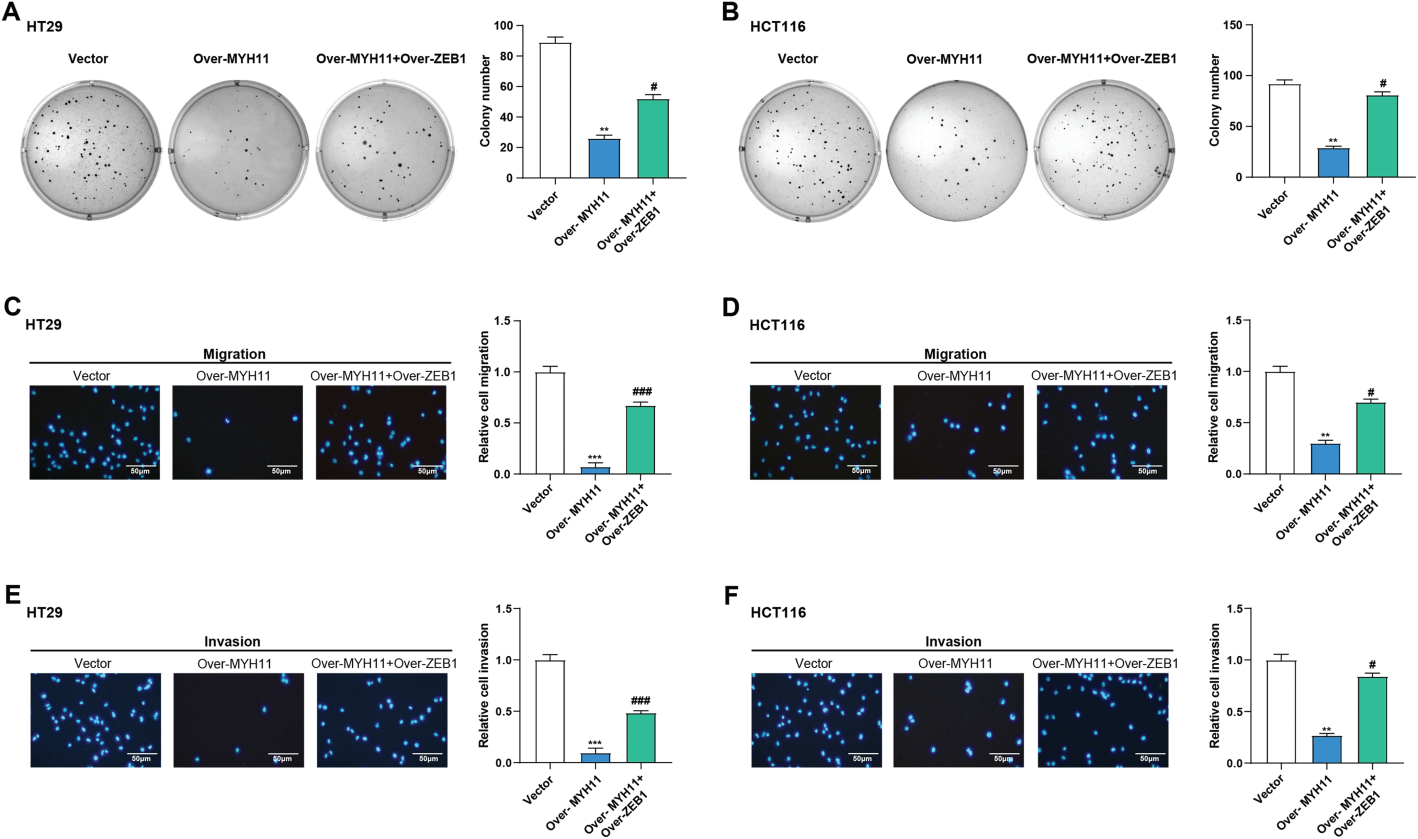
*MYH11* regulates the cellular behavior of CRC cells through *ZEB1*. (**A**,**B**) Colony formation assay detected the colony formation ability of HT29 (**A**) and HCT116 (**B**) cells overexpressing *MYH11* or co-overexpressing *MYH11* and *ZEB1*. (**C**,**D**) Transwell detected the migration abilities of HT29 (**C**) and HCT116 (**D**) cells overexpressing *MYH11* or co-overexpressing *MYH11* and *ZEB1*. (**E**,**F**) Transwell detected the invasion abilities of HT29 (**E**) and HCT116 (**F**) cells overexpressing *MYH11* or co-overexpressing *MYH11* and *ZEB1*. Scale bar: 50 μm. **p < 0.01, ***p < 0.001 vs. Vector. ^#^*p* < 0.05, ^###^*p* < 0.001 vs. over-*MYH11*. CRC: Colorectal cancer

Subsequently, we continued investigating the possible mechanism whereby *MYH11* combined with *ZEB1* affects the cell behavior of CRC cells. As demonstrated by colony formation experiments, overexpression of *MYH11* significantly reduced the cell viability of CRC cells. However, the combined overexpression of *MYH11* and *ZEB1* partially rescued this effect (Fig. 9A,B). In the Transwell assay, CRC cells overexpressing *MYH11* exhibited considerably lower migration and invasion abilities compared to the control, and this inhibitory effect was partially reversed when ZEB1 was overexpressed in conjunction with *MYH11* (Fig. 9C–F). These findings highlight that *MYH11* can regulate the behavior of CRC cells through *ZEB1*.

## 4 Discussion

CRC is a highly prevalent gastrointestinal tract malignancy, characterized by a complex interplay between genetic and environmental factors [35]. Early-stage symptoms are often subtle, making detection challenging, and advanced stages are associated with poor prognosis and high mortality [4]. Uncovering the key molecular mechanisms driving tumorigenesis is crucial for the targeted therapy of CRC. Thus, several studies have explored the roles of various genes in CRC [36–38]. In this study, we conducted a thorough bioinformatics analysis on the TCGA-READ and GSE123390 datasets. *MYH11* was identified as a hub gene based on our results, which showed that it was considerably downregulated in tumor samples as compared to normal samples. Our study aimed to investigate the role of *MYH11* in CRC progression, particularly its effects on EMT and cell behavior, and to explore its potential regulation by the EMT transcription factor *ZEB1*.

As a member of the myosin family, MYH11 plays a crucial role in cell mobility and muscle contraction. It is closely related to functions such as cell migration, adhesion, control of cell shape, and membrane transport [39]. Truncating mutants of *MYH11* may influence the energy balance and motility of cancer cells, since they have been shown to display elevated ATPase and motility activities in cancer [40]. Several studies have identified *MYH11* as a potential key gene in CRC through bioinformatics analysis. Wang et al. have discovered *MYH11* mutations in CRC. Furthermore, it has been found that in stage II and III colorectal cancer, a poor prognosis was linked to downregulated *MYH11* expression [41]. This is consistent with our findings that *MYH11* is downregulated in CRC cells. Overexpression study results demonstrated that overexpression of *MYH11* inhibited the proliferation, invasion, and migration of CRC cells, further supporting its potential as a tumor inhibitor in CRC.

A biological process known as EMT enables epithelial cells to acquire mesenchymal traits, thereby promoting enhanced migration and invasiveness. Due to the relevance of EMT to cancer cell metastasis, many studies related to cancer treatment have attempted to target EMT. EMT is also critical for CRC metastasis and has therefore been extensively studied. Our study further explores the impact of *MYH11* overexpression on EMT dynamics in CRC cells. Results suggest that *MYH11* not only inhibits cell proliferation but also plays a vital part in suppressing EMT in CRC cells. This finding is consistent with numerous studies that have demonstrated the potential of inhibiting EMT in CRC cells by targeting specific genes to further suppress cancer progression. Dang et al. concluded that *P4HA2* can induce EMT in CRC cells, which promotes cancer progression [42]. *S100A8* promotes EMT in CRC cells under the action of the TGF-β/USF2 axis [43]. In CRC, *SNAIL* was also observed to induce the secretion of CXCL2, promoting EMT and lung metastasis of CRC cells [44]. By modulating these key EMT markers, *MYH11* may serve as a crucial regulator in maintaining epithelial characteristics, thereby hindering tumor invasion and metastasis.

In this study, we also investigated the functional impact of *ZEB1* on the behavior of CRC cells. Our results confirmed that *ZEB1* overexpression significantly promotes EMT in addition to CRC cell viability and behavior. By triggering EMT, the transcription factor *ZEB1* promotes tumor invasion and metastasis. It is essential for many types of cancer, but especially for certain types including liver and non-small cell lung cancer [45,46]. According to studies, a poor patient prognosis is linked to increased *ZEB1* expression, which also positively correlates with cancer invasion and metastatic potential [47]. Wei et al. demonstrated that *ZEB1* suppresses E-cadherin expression, thereby contributing to EMT [48]. Additionally, several reports link *ZEB1* to CRC progression. For example, *ZEB1* is a direct transcriptional target of *FOXK2*, which induces EMT in CRC cells through *ZEB1* activation [49]. Similarly, *RHBBD1* promotes CRC metastasis by activating the Wnt signaling pathway and its downstream target *ZEB1* [50]. According to other research, *DAPK1* downregulation activates *ZEB1*, which in turn enhances stemness and EMT in CRC [51]. Notably, this study reveals that MYH11 interacts with ZEB1 to regulate the proliferation, migration, and invasion of CRC cells. These findings suggest that *ZEB1* not only contributes to CRC aggressiveness but is also a key mediator of the regulatory effects of *MYH11*. Our findings suggest that while *MYH11* overexpression strongly suppresses *ZEB1* and EMT, the partial restoration of mesenchymal markers upon *ZEB1* co-overexpression hints at a potential compensatory mechanism where elevated *ZEB1* may counteract *MYH11*’s inhibitory effects. Unlike ZEB1, which directly drives EMT through transcriptional repression of epithelial markers, *MYH11* likely acts upstream, modulating *ZEB1* activity without evidence of direct feedback to itself. This asymmetric regulation implies that *MYH11*’s role is dependent on *ZEB1* suppression, whereas *ZEB1* overexpression can bypass *MYH11*-mediated inhibition, pointing to distinct hierarchical control mechanisms. Further studies are needed to validate whether *ZEB1* actively feedback-regulates *MYH11*. Although *MYH11* is mainly expressed in smooth muscle cells, it has been reported to be dysregulated in epithelial cancers. For example, Wang et al. [41] observed that *MYH11* is downregulated in advanced CRC tissues, which is associated with a poor prognosis, indicating its potential tumor-suppressive role in epithelial contexts. In our study, *MYH11* overexpression in CRC cells suppressed EMT, as evidenced by increased E—cadherin and decreased N-cadherin, vimentin, and ZEB1. This is consistent with the finding that *MYH11* loss in smooth muscle cells disrupts cytoskeletal integrity and promotes a mesenchymal phenotype. Additionally, *MYH11* overexpression in CRC cells reduces cell viability, possibly due to its role in promoting epithelial differentiation and causing cells to exit the cell cycle. For instance, *MYH11* overexpression in vascular smooth muscle cells induces contractile differentiation and reduces proliferative capacity [13]. Similarly, in CRC cells, *MYH11* may induce a less aggressive, differentiated state, characterized by restored E-cadherin and suppressed ZEB1. We speculate that *MYH11* overexpression enforces a “partial differentiation” program, restricting tumorigenic potential. Future studies will evaluate differentiation markers like cytokeratin-20 and CDX2 to test this hypothesis. By targeting *ZEB1*, *MYH11* demonstrates its ability to regulate EMT, laying a theoretical foundation for investigating whether *MYH11* could serve as a therapeutic target in CRC.

Despite the significant findings of our study, a substantial gap remains in understanding the intricate mechanisms by which *MYH11* exerts its effects in cancer biology. Our study highlights the inhibitory role of *MYH11* in CRC progression, particularly through its regulation of *ZEB1* and EMT. However, we did not investigate other EMT-regulating transcription factors, such as Snail, Slug, and Twist. Future studies should examine their expression and involvement when *MYH11* is overexpressed in CRC cells for a fuller understanding of *MYH11*-regulated EMT. Additionally, we did not create stable cell lines overexpressing *MYH11* and *ZEB1*, which would be useful for long-term effect studies. Future research should establish such lines to better assess the sustained impacts of these proteins on CRC. Moreover, the detailed mechanisms and broader impacts of *MYH11* in cancer biology, including its interactions with other signaling molecules and its effects on processes like angiogenesis and immune evasion, remain unclear. Additionally, the therapeutic potential of targeting *MYH11* in cancer treatment is largely unexplored. Further research is needed to elucidate the *in vivo* role of *MYH11* and to validate its potential as a therapeutic target.

## 5 Conclusion

In conclusion, our study elucidates the significant role of *MYH11* as a key regulator in CRC progression and underscores the importance of molecular mechanisms in CRC. Compared to normal tissues, *MYH11* is markedly downregulated in CRC tissues, and its overexpression significantly inhibits CRC cell proliferation, migration, and invasion by downregulating EMT markers and targeting ZEB1, a key promoter of malignant cell behavior. These findings highlight the critical involvement of *MYH11* in CRC pathophysiology and its potential as a novel therapeutic target. However, this study has limitations, including a relatively small sample size, which may affect the generalizability of the results. Future studies will include immunohistochemistry experiments to verify the expression and distribution of target proteins in CRC tissues. Priority will be given to *in vivo* models to validate *MYH11*’s antitumor effects, explore its synergistic potential with existing therapies, and further dissect the molecular interactions underlying its regulatory mechanisms. Such efforts could uncover new therapeutic strategies and deepen our understanding of metastatic processes in CRC and other malignancies.

## Supplementary Materials



## Data Availability

The datasets used and analyzed during the current study are available from the corresponding authors upon reasonable request.

## Supplementary Materials

The supplementary material is available online at https://www.techscience.com/doi/10.32604/or.2025.063501/s1.
