# Supplementary material for: MYH11 Suppresses Colorectal Cancer Progression by Inhibiting Epithelial-Mesenchymal Transition via ZEB1 Regulation
Source: Oncol Res. 2025 Aug 28;33(9):2379–98. doi: 10.32604/or.2025.063501 (PMC12408857; doi:10.32604/or.2025.063501)
Supplement: Supplementary file 1 [file OncolRes-33-63501-s001.docx]

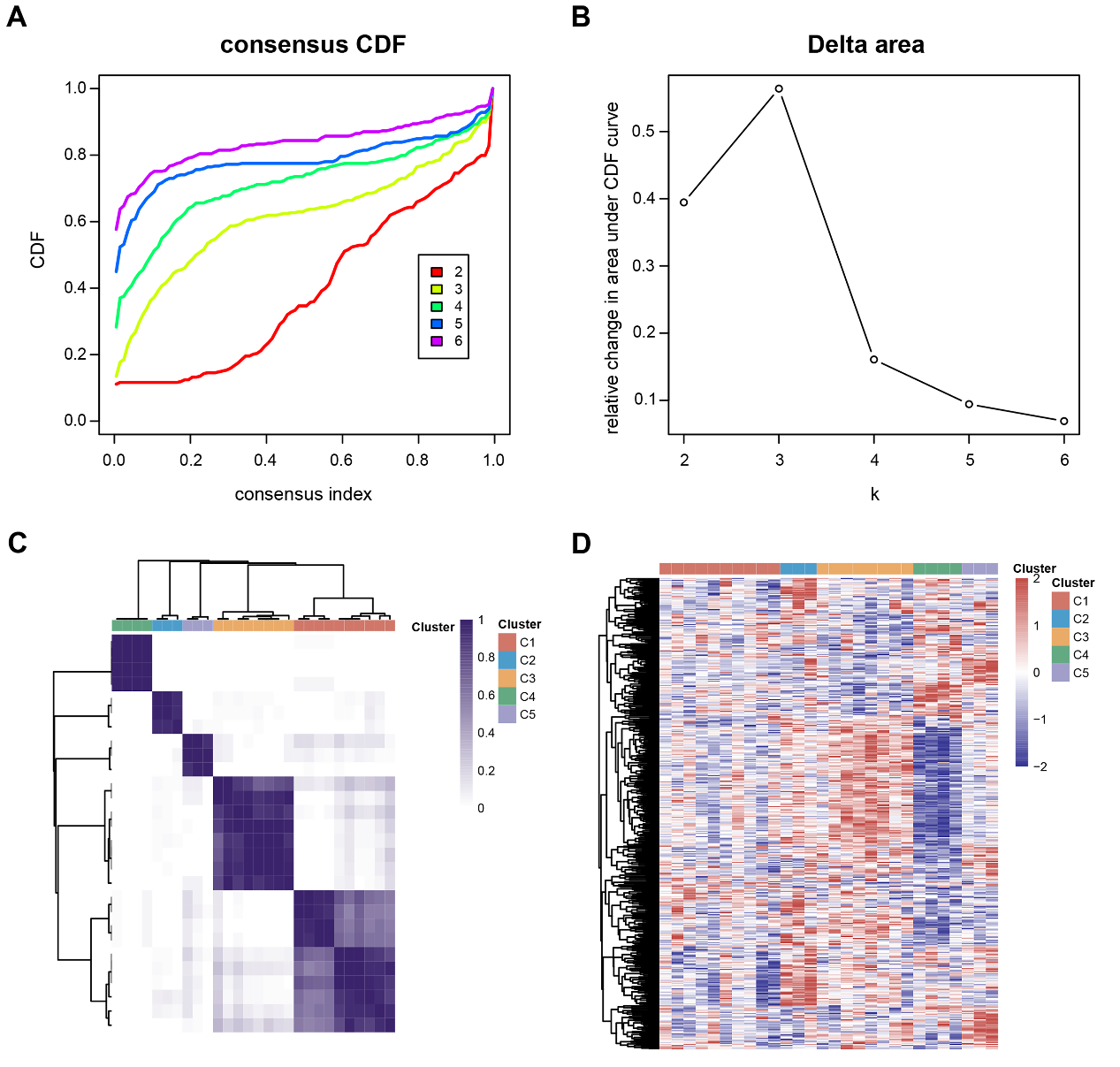


**Supplementary Figure S1. Identification of molecular subtypes in CRC based on DEGs from the GSE123390 dataset.**

(A) Cumulative distribution function (CDF) curves for k = 2 to k = 6 clusters. The CDF curve becomes smoother and more saturated as the number of clusters increases, indicating increasing stability.

(B) Delta area plot showing the relative change in the area under the CDF curve for each k. It is most significant at k = 3, and the curve flattens at k = 5, indicating that it is the optimal number of clusters.

(C) Consensus heatmap for k = 5. Columns of different colors represent different categories (C1-C5).

(D) Heatmap of 441 genes in the C1 to C5 groupings. Columns represent samples, grouped by cluster assignment (C1 to C5), and rows represent genes. Red and blue represent high and low expression levels, respectively.

**
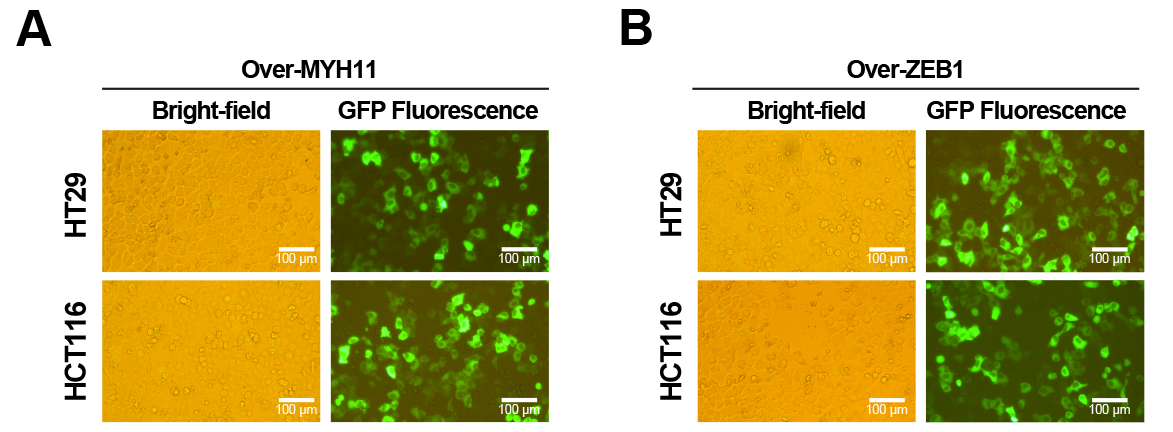
**

**Supplementary Figure S2. Fluorescence images of transfection efficiency for MYH11 and ZEB1 overexpression plasmids in HT29 and HCT116 cell lines.**

(A) HT29 and HCT116 cells were transfected with pcDNA-3.1-GFP-MYH11, and green fluorescence was observed under a fluorescence microscope after 48 hours, indicating successful transfection. Scale bar: 100μm.

(B) HT29 and HCT116 cells were transfected with pcDNA-3.1-GFP-ZEB1, and transfection efficiency was similarly evaluated based on GFP fluorescence. Scale bar: 100μm.


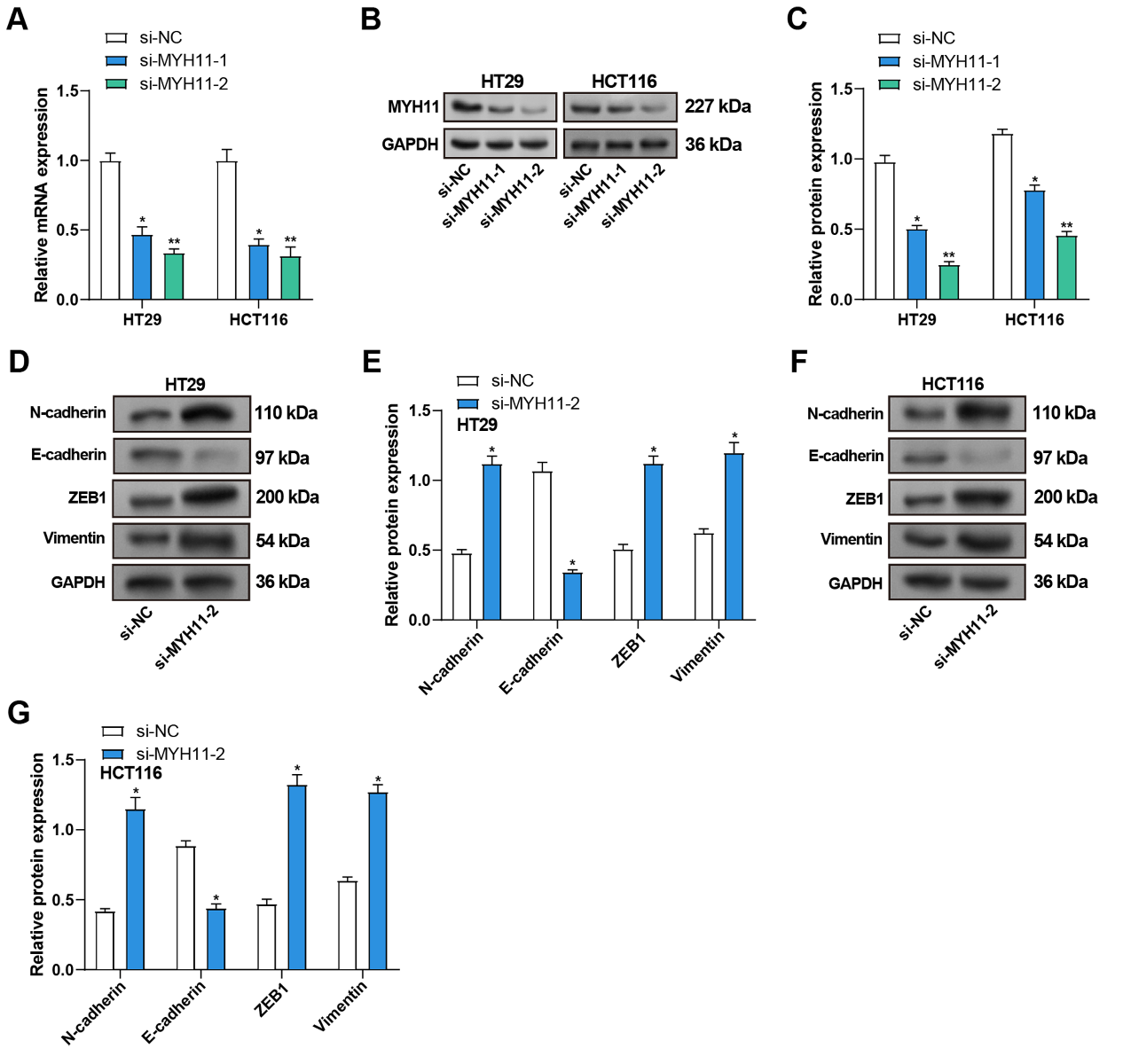


**Supplementary Figure S3. Knockdown of *MYH11* promotes EMT in CRC cells.**

(A) qRT-PCR detected *MYH11* mRNA expression in CRC cells with *MYH11* knockdown. (B and C) WB detected the protein expression level of MYH11 in CRC cells with *MYH11* knockdown, and performed quantitative analysis. (D and F) WB detected the protein expression levels of EMT-related proteins (E-cadherin, N-cadherin, Vimentin, and ZEB1) in HT29 (D) and HCT116 (F) cells with *MYH11* knockdown, and quantitative analysis (E and G). **p*<0.05, ***p*<0.01. CRC: Colorectal cancer, WB: Western blotting, EMT: Epithelial-mesenchymal transition.


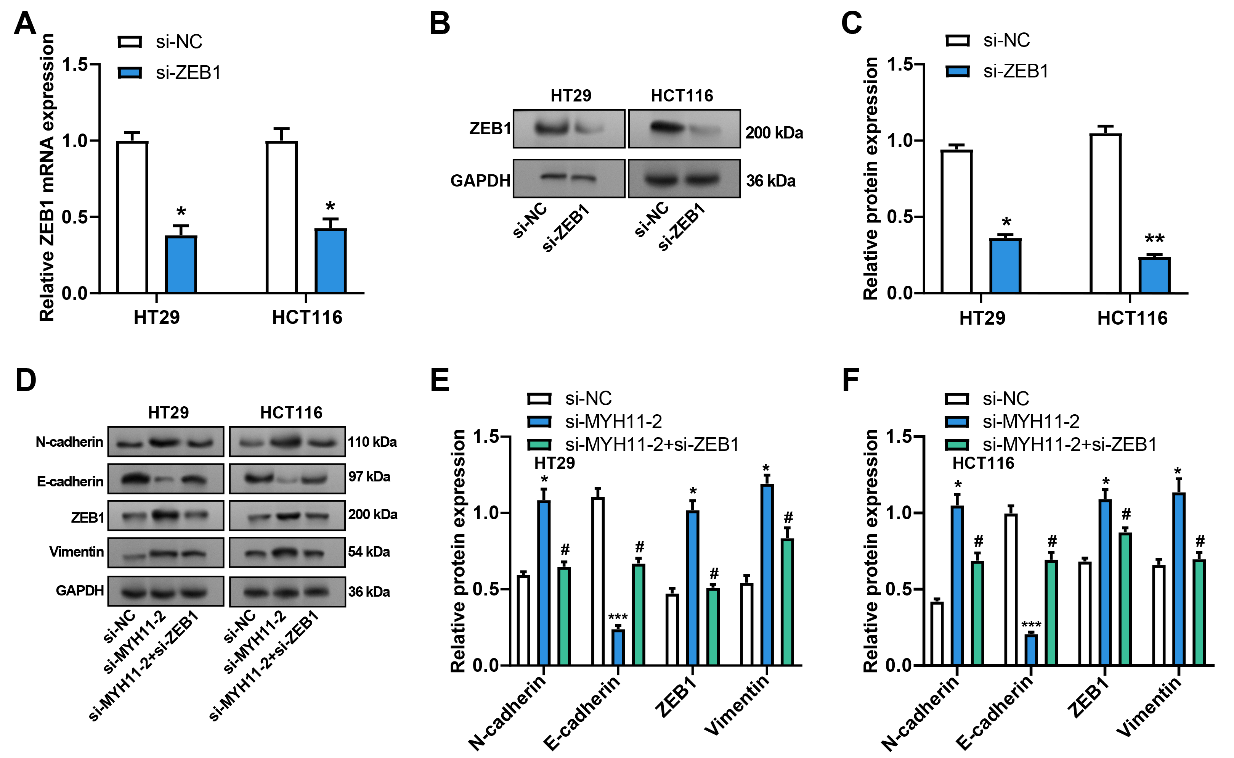


**Supplementary Figure S4. The co-knockdown of *MYH11* and *ZEB1* alters EMT-related proteins expression in HT29 and HCT116 cells.**

(A) qRT-PCR detected *ZEB1* mRNA expression in CRC cells with *ZEB1* knockdown. (B and C) WB detected the protein expression level of ZEB1 in CRC cells with *ZEB1* knockdown, and performed quantitative analysis. (D) WB analysis of EMT markers (E-cadherin, N-cadherin, ZEB1, and Vimentin) in HT29 and HCT116 cells transfected with si-*MYH11* alone or co-transfected with si-*MYH11* and si-*ZEB1*. (E) Quantitative analysis of protein expression levels in HT29 cells. (F) Quantitative analysis of protein expression levels in HCT116 cells. **p*<0.05, ***p*<0.01, ****p*<0.001 *vs.* si-NC. ^#^*p*<0.05. CRC: Colorectal cancer, WB: Western blotting, EMT: Epithelial-mesenchymal transition.
